# Efficacy of the Otago-Exercise-Programme to reduce falls in community-dwelling adults aged 65-80 when delivered as group or individual training: Non-inferiority-clinical-trial

**DOI:** 10.1186/s12912-024-02310-3

**Published:** 2024-10-01

**Authors:** Laura Albornos-Muñoz, Joan Blanco-Blanco, María Ángeles Cidoncha-Moreno, Eva Abad-Corpa, Araceli Rivera-Álvarez, Rosa María López-Pisa, José Manuel Caperos, Otago Project Working Group Consortium, Laura Albornos-Muñoz, Joan Blanco-Blanco, María Ángeles Cidoncha-Moreno, Eva Abad-Corpa, Araceli Rivera-Álvarez, Rosa María López-Pisa, María Pilar Rodríguez Baz, Ana Bays Moneo, Laura Pruneda González, Dawn A Skelton, Chris Todd, Rebecca Townley, Pedro Luis Pancorbo Hidalgo, Oscar Caño Blasco, María Cristina Solé Agusti, Manuel Rich-Ruiz, Ana Covadonga González Pisano, Jerónima Miralles Xamena, María Consuelo Company Sancho, María Teresa Moreno-Casbas, María Teresa Moreno-Casbas

**Affiliations:** 1 Nursing and Health Care Research Unit (Investén-isciii), Research Network on Chronicity, Primary Care and Prevention and Health Promotion (RICAPPS), Instituto de Salud Carlos III, Madrid, Spain; 2 Comprehensive Care and Health Services Programme (PhD candidate), Department of Nursing and Physiotherapy, University of Lleida, Lleida, Spain; 3 Department of Nursing and Physiotherapy, Group for the Study of Society Health Education and Culture, GESEC, University of Lleida; Health Care Research Group, GRECS Biomedical Research Institute of Lleida IRB-Lleida; Biomedical Research Network Center in Frailty and Healthy Aging (CIBERFES), Lleida, Spain; 4 Bioaraba Health Research Institute, Vitoria, Spain, Basque Health Service, General Head Office of Osakidetza Subdirection of Nursing, Vitoria, Spain. Primary Care and Prevention and Health Promotion (RICAPPS), Academy of Nursing Sciences of Bizkaia, Bilbao, Spain; 5 University of Murcia-SMS, Investén-isciii, Biomedical Research Network Center in Frailty and Healthy Aging (CIBERFES), IMIB-Arrixaca, Murcia, Spain; 6 Abrantes Primary healthcare center, Dirección Asistencial Centro, Madrid, Spain; 7 Institut Català de la Salut, Barcelona, Spain; 8 UNINPSI, Psychology Department, Pontificia Comillas University, Madrid, Spain; 9 Nursing and Health Care Research Unit (Investén-isciii), Biomedical Research Network Center in Frailty and Healthy Aging (CIBERFES), Instituto de Salud Carlos III, Madrid, Spain; 10 Instituto de Salud Carlos III, Madrid, Spain; 11 Department of Health Sciences, Public University of Navarra, Navarra, Spain; 12 University of Oviedo, Oviedo, Spain; 13 Reseach Centre for Health (ReaCH), School of Health & Life Sciences, Institute of Applied Health Research, Glasgow Caledonian University, Glasgow, UK; 14 School of Health Sciences, Faculty of Biology, Medicine & Health, The University of Manchester, Manchester, M13 9PL UK; 15 Manchester University NHS Foundation Trust, Manchester, M13 9WL UK; 16 Later Life Training Ltd, Northumberland, UK; 17 Department of Nursing, Faculty of Health Science, University of Jaén, Jaén, Spain; 18 Murcian Health Service, Murcia, Spain; 19 Instituto Maimonides de Investigacion Biomédica (IMIBIC). Universidad de Córdoba. Hospital Universitario Reina Sofía, Biomedical Research Network Center in Frailty and Healthy Aging (CIBERFES), Córdoba, Spain; 20 Service of Health of the Principality of Asturias, University of Oviedo, Oviedo, Spain; 21 Primary Care Research Unit of Mallorca, Baleares Health services-IbSalut, Palma, Spain; 22 Health Promotion Service. Directorate General for Public Health. Canary Islands Health Service, Hermigua, Spain

**Keywords:** Accidental Falls, Exercise Therapy, Clinical Trial, Primary Care, Otago Exercise Programme

## Abstract

**Background:**

The Otago Exercise Programme is an effective intervention for falls prevention. However, there is limited evidence in relation to studies that compare efficacy for falls prevention when delivered Otago Exercise Programme in a group or individual format in a primary care context.

**Objective:**

To compare the Otago Exercise Programme delivered as a group vs. individual format for community dwelling older adults, over a one year period. The hypothesis was that neither format would be inferior to the other.

**Methods:**

**Design:**

A four-year multicentre, randomized, non-inferiority clinical trial, with two arms— Otago Exercise Programme group training and individual Otago exercise training. Setting(s): 21 primary healthcare centers. Participants: A sample size of 728 participants was established. Participants were aged between 65 and 80 years; living in the community; able to walk independently; and agreed to take part in the study and provided signed informed consent. Intervention: The Otago Exercise Programme was delivered mainly by nurses in primary care, with five face to face sessions, and a reinforcement 6 months later. Participants were encouraged to exercise at home between face to face sessions. Data collection: at baseline and after 6 and 12 months from October 2017 to 2020. Primary outcome: people who reported at least one fall. Secondary outcomes: number of falls, cause of falls, consequences and assistance, adherence and satisfaction. Group allocation was blinded to the researchers involved in analysis. Reporting: Consolidated Standards of Reporting Trials recommendations for the Statement for Randomized Trials of Nonpharmacologic Treatments.

**Results:**

Eight hundred twenty-seven participants were randomized (226 were allocated in group training and 272 in individual training). The analysis of the proportion of people who reported at least one fall and number of falls showed no differences between individual and group training. Assessment of the equivalence between the interventions at 12 months showed that the confidence interval for the difference of people who reported at least one fall was found to be within the equivalence limit of 10% considered. However, in those participants with a previous history of falls, group format showed potentially greater benefit. The participants in individual training presented higher scores on the Exercise Adherence Rating Scale test. No differences were found in satisfaction between the groups.

**Conclusions:**

The group Otago Exercise Programme is equivalent to individually delivered Otago Exercise Programme in terms of prevention of falls over a 12-month follow up. Adherence was higher in individual training. Implications: Healthcare professionals could offer either Otago Exercise Programme format dependent on patient preference and be confident that that standardized intervention provides patient benefit.

**Trial Registration:**

ClinicalTrials.gov (NCT03320668). Data registration 31/10/2017.

**Supplementary Information:**

The online version contains supplementary material available at 10.1186/s12912-024-02310-3.

## Background

A fall can be defined as an event that results in a person coming to rest inadvertently on the ground or floor or another lower level [1]. Globally, falls are considered a major problem in public health due to their frequency, morbidity, mortality and related costs [2]. In addition, they are among the main geriatric syndromes and have a great impact on elder adult prognoses, as they are associated with frailty and functional decline [3]. Within Europe an average of 35,848 adults aged 65 years or over die each year due to fall-related injuries, and this figure is believed to be an underestimation of the real number of fall-related deaths [4]. In the community setting in particular, it is estimated that 30% of people aged 65 and over suffer falls each year [5, 6] and 15% of these individuals suffer more than one fall, with increasing rates of disability and mortality [7]. These events can have physical and emotional impacts on older people and worsen their quality of life [8].

Falls are not an inevitable result of aging, considering that systematic reviews of fall intervention studies have established that prevention interventions can reduce falls [9]. In 2015, the Centers for Disease Control and Prevention (CDC) published the third edition of the compendium of effective fall prevention interventions [10]. The Otago Exercise Programme was included among the exercise interventions shown to be effective at preventing falls. This intervention was developed as a programme of progressive exercise that included strength and balance components and other studies that have shown to be efficacious at reducing falls in community-dwelling older people [9–17]. The Otago Exercise Programme was originally based on four clinical trials demonstrating that it was a well-designed, effective programme for reducing falls and increasing strength and balance in older people living independently whether they had suffered a fall or not [13–17]. To deliver this exercise program, personnel must be trained [11]. Later Life Training deliver a model of cascade training which was implemented in 11 countries in Europe during the European Project Prevention of Falls Network for Dissemination (ProFouND) [18], and used a standardized protocol for group and individual delivery [19]. According to this model, Cascade Trainers were trained to prepare other heathcare professionals as Otago Exercise Programme Leaders, that then delivered the Program to the participants selected.

There are several effective fall prevention interventions related to exercise programs such as Otago Exercise Programs, however there are limited evidence in relation to its delivery formats. A systematic review of fall prevention interventions in the community concluded that group and home exercise programs not only reduced falls, relating to rate or number of falls, or number of participants sustaining at least one fall during follow up but also the risk of falling [5]. A more recent review “Exercise for preventing falls in older people living in the community” provide aligned conclusions, showing that exercise interventions have been found to be effective when delivered in a group‐based setting or on an individual basis, however the optimal features of successful fall prevention exercise programmes are not yet clear [20]. According to more recent secondary research studies, extensive further research is necessary regarding the prevention of falls in the clinical setting, in group versus individual modalities of exercise interventions and in the primary care setting.

The aim of this study was to compare people who reported at least one fall of group vs individual Otago Exercise Programme in community dwelling people aged 65 to 80 years in primary healthcare context. Secondary aims: To compare number of falls, cause of falls, consequences and assistance, adherence and satisfaction of the Otago Exercise Programme as a group or individual formats in community dwelling people aged 65 to 80 years in primary healthcare context.

The hypothesis is that the delivery of the Otago Exercise Programme in a group is not inferior to the delivery of the Programme in an individual format in terms of people who reported at least one fall.

## Methods

A multicenter, simply blinded, randomized, noninferiority, one-year follow-up clinical trial with two arms (Otago Exercice Programme group and individual) was performed. The study took four years. Primary outcome:people who reported at least one fall. Secondary outcomes: number of falls, cause of falls, consequences and assistance, adherence and satisfaction. The protocol of the study was published previously [21]. The study has been registered at ClinicalTrials.gov (NCT03320668). Data collection was completed at baseline and after 6 and 12 months from October 2017 to 2020, This paper was presented using the Consolidated Standards of Reporting Trials (CONSORT) recommendations for the Statement for Randomized Trials of Nonpharmacologic Treatments [22] (see Additional file 1).

### Study setting and sampling

Primary healthcare centers (*n*=21), from 8 regions, and belonged to the same basic health zone. Participation was offered to all people that visit the primary healthcare center during the recruitment period and met the inclusion criteria.

### Sample size

For the sample size estimation, we assumed that 40% of the participants aged 65 years or older would fall during a 1-year period [6, 23]. A sample size of 364 participants was calculated for each of the two study groups (group vs. individual). This was based on unilateral contrast with a type I error equal to 0.025 and a 1-beta power equal to 0.80 in a unilateral contrast agent, assuming a decrease in falls of 15% and setting a non-inferiority limit of 10%. Based on the findings of previous studies, a loss of 10% to follow-up was assumed.

### Inclusion and/or exclusion criteria

Inclusion criteria

People:

1. aged between 65 and 80 years at the time of study participation was offered;
2. that were living in the community;
3. that were able to walk independently;
4. and that agreed to take part in the study and provided signed informed consent.

Exclusion criteria

1. People who had been living on permanent property in the area covered by the primary healthcare center for <9 months or who had a life expectancy of <9 months;
2. Moderate or severe cognitive impairment according to Mini Mental State Examination tool (MMSE) (these criteria were modified from the protocol);
3. People with sight or hearing impairment that would prevent them following the intervention;
4. Absolute contraindications to performing physical exercise; and
5. People who were already participating in another clinical trial, research study or exercise programme where they performed balance and strength activities similar to the Otago Exercise Programme exercises.

Exclusion criteria (2), (3) and (4) were established according to diagnoses in the individual’s medical history.

### Study dropout criteria

1. Revocation of informed consent
2. Change in health area, change to institutionalized status or death
3. Changes in clinical status that affected the continuation of the exercise program
4. The occurrence of muscle pain, joint pain, chest pain, shortness of breath or fall while performing the prescribed exercises required a new joint assessment of the reference primary healthcare team to determine study dropout.

### Study Intervention common description for two arms

The clinical trial consisted of two arms. In both, the Otago Exercise Programme was delivered in the primary healthcare center. In one arm, the test was carried out individually, and in the other, the test was delivered in a group format. Both interventions were carried out by a health professional who had received training as an Otago Exercise Programme Leader (nurse/physiotherapist) [18, 19]. Ankle weights were used for strengthening exercises. The progression of both strength and balance exercises was established in three levels of difficulty: beginners, intermediate and advanced level. Progression in strengthening exercises was established by increasing the number of repetitions and weight, and in balance exercises by reducing supports. The progression criteria were the same in both groups; the possible progression from the second week, in addition to the change in level, was achieved by adding more weight to the ankles, increasing the number of repetitions or reducing support. The program included five face to face sessions, distributed according to following information:

- 1^st^ week: training and prescription of the Otago Exercise Programme exercises – beginners level.
- 2^nd^ week: training and prescription of the Otago Exercise Programme exercises – beginners level, with progression if applicable.
- 4^th^ week: training and prescription of the Otago Exercise Programme exercises - intermediate level, with progression if applicable.
- 8^th^ week: training and prescription of the Otago Exercise Programme exercises - advanced-level.
- Reinforcement session at six months: All-level, with progression if applicable.

According to the protocol, the intervention was completed if the participant carried out all sessions according to progression criteria. In addition to the sessions delivered and supervised at the primary healthcare center, both groups were encouraged to complete 30 minutes of exercise twice a week, or perform them by including them in their daily routine. In case the participant did not attend all session, the participant dropped out of the study however the treatment/referrals continued.

Each participant received a diary to collect the exercise performance, audiovisual and written support material with instructions for each prescribed exercise, along with the necessary equipment to perform the muscle-strengthening exercises (ankle weights). Reminders were given for appointments, assisted by an alert system with messages or telephone calls according to a predetermined telephone interview protocol. Prompts to continue to exercise at home occurred every two weeks during the first three sessions and every four weeks from the eighth week whenever the call did not coincide with a face to face session. The diary and protocol of follow up was used by healthcare professionals to verify the quality of the prescribed exercises.

Participants were informed that they should continue to perform their normal routines with respect to physical or leisure activities without modification and add the Otago Exercise Programme to their schedules, as these exercises do not replace any of the activities that they previously carried out.

### Individual training

The health professional conducted individualized face to face exercise sessions with each participant in five sessions at the primary healthcare center: in the 1st, 2nd, 4th and 8th weeks and in a reinforcement session at 6 months. The first visit lasted one hour, and the remaining sessions were 30 minutes each.

### Group training

For the purposes of this study, group training was defined as a group consisting of 6 or more participants up to a maximum of 12. In terms of viability, this group could be formed by participants included in the clinical trial and/or people who were not included.

The health professional, supported by another Otago Exercise Programme Leader (nurse/physiotherapist), both of whom had received Otago Exercise Programme Leader training, performed the group training at weeks 1, 2, 4 and 8 and a reinforcement session at 6 months. The first workshop lasted one hour, and the remaining sessions were 45 minutes each.

### Randomization

The assignment of participants to each of the study groups was conducted in a stratified, random, individual manner. In each center, the randomized sequence comprises hidden swapping blocks of two, four and six. Each center received their sequence of randomization in closed and opaque envelopes from the trial’s Coordinator Centre.

### Blinding

It was not possible to blind the study participants or the professionals who carried out the intervention in terms of the intervention (group vs. individual). Group allocation was blinded to the researchers involved in analysis**.**

### Variables

Primary outcome: people who reported at least one fall.

Secondary outcomes: number of falls, cause of falls, consequences and assistance, adherence [24] and satisfaction. Adverse events were included in registered consequences.

Explanatory variable: assigned group (group and individual training).

The secondary explanatory variables included sociodemographic (age, sex, education, civil status) and clinical (weight, height, BMI, cognitive status, Tinetti (risk of falls assessment), fear of falling, Barthel Index, Lawton Instrumental Activities of Daily Living, frailty, comorbidity and polypharmacy) [21].

### Recruitment and data collection

#### Recruitment

Recruitment was actively performed by the health professionals selected in each basic health area among those who met the inclusion criteria and who were treated at their usual consultations. This recruitment was carried out consecutively among people with scheduled appointments on specific days. According to the protocol, these periods of inclusion could be interrupted during the influenza vaccination campaign or by the occurrence of any other eventuality, subject to the agreement of the researchers.

#### Data collection

Data registration were effective since 31^th^ October 2017, and was reported at baseline and after 6 and 12 months.

Each professional had a field notebook for including information about the recruitment process in which the details of the excluded people were recorded with respect to the reasons for exclusion.

Each professional collected data for all variables in each face to face session and documented these in the participant medical record and in the Data Collection Form for the Clinical Trial. Additionally the participants had an exercise diary to collect the exercises performed at home, to be reviewed by the health professionals and do follow up of the intervention.

### Data analysis

A descriptive analysis stratified by intervention arm was carried out for sociodemographic, sociological and clinical variables and information on falls: frequencies and percentages, mean and standard deviation. To analyze the differences by group, the Student’s t-test or the Mann-Whitney U test was used according to the nature of the continuous variables after checking their distribution. For qualitative variables, the Chi-square test was employed.

The methods proposed by Westlake and Lakens [25–27] were used to assess the equivalence between the interventions in the primary outcome measure (people who reported at least one fall), calculating the 90% confidence interval for the difference in the proportion of falls between the study groups. The proportions of participants who experienced falls were compared at 6 and 12 months. An equivalence margin of 10% in the proportion of falls was used to consider the treatments as equivalent.

To evaluate differences in post-intervention in relation to baseline between the two intervention arms, the response variables were analyzed as follows: a mixed effect logistic model was used to model the people who reported at least one fall, a mixed effect Poisson model was used to model the number of falls, mixed effect logistic model was used to model the cause of falls, consequences and assistance, and mixed linear model was used to model the adherence. Interactions between intervention arms and follow-up periods, baseline, 6 months and 12 months were considered. End-of-treatment satisfaction in the two study groups was compared through Student’s t test for independent measures. A confidence interval of 95% was utilized for all comparisons, and analyses were carried out using the Jamovi program [28].

## Results

**Fig. 1:**
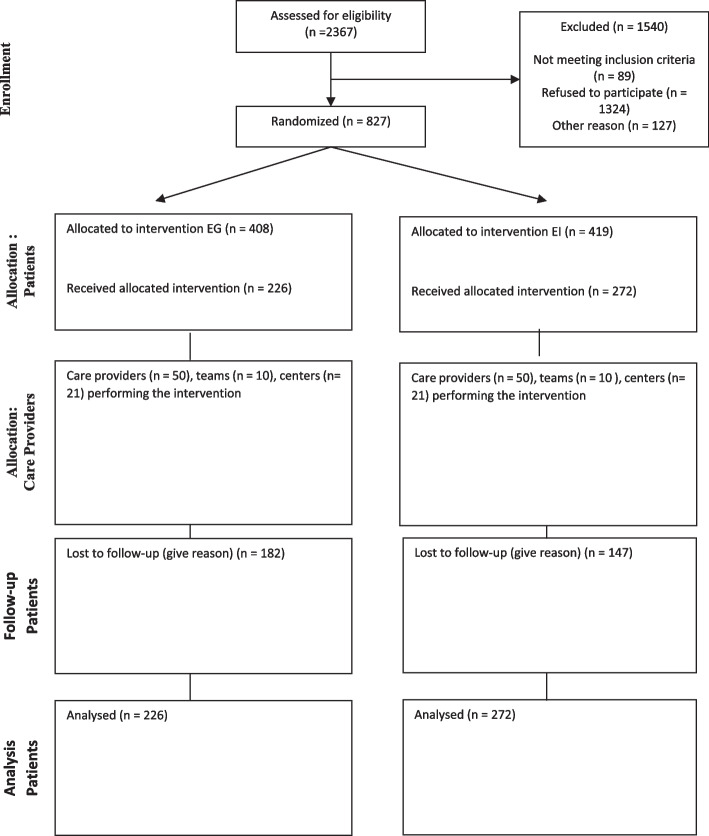
Modified CONSORT flow diagram for individual randomized controlled trials of nonpharmacologic treatments. An extra box per intervention group relating to care providers and centers has been added. IQR = interquartile range; max = maximum; mi*n =* minimum

A total of 2367 participants were recruited; 827 met the inclusion criteria, agreed to participate by signing informed consent forms and were randomized. Of the 827 participants who opted to participate, 747 began the interventions. A total of 498 participants completed the intervention at 12 months, 226 in the group and 272 in the individual Otago Exercise Programme (Fig. 1). Table 1 shows the analysis of sociodemographic (age, sex, education, civil status) and clinical (weight, height, Body Mass Index (BMI), cognitive status, Tinetti (risk of falls assessment), fear of falling, Barthel Index, Lawton Instrumental Activities of Daily Living, frailty, comorbidity and polypharmacy) variables for both groups at baseline. No statistically significant differences were found between the groups at baseline.

**Table 1:** Analysis of sociodemographic and clinical variables

*Numeric variables*	Individual training		Group Training		Student’s t test
	Mean	SD	Mean	SD	
Age	71.6	4.1	72.1	4.2	*p =* 0.171
Weight	75.5	13.1	74.4	12.7	*p =* 0.338
Height	160.2	9.1	159.3	8.3	*p =* 0.291
BMI	29.4	4.5	29.3	4.5	*p =* 0.746
Cognitive	28.4	1.8	28.4	1.8	*p =* 0.956
Tinetti	32.1	3.9	31.7	4.2	*p =* 0.304
Fear of falling	21.4	6	21.2	5.6	*p =* 0.794
Barthel	99.2	2.5	98.9	3.3	*p =* 0.173
Lawton	7.8	0.7	7.8	0.7	*p =* 0.599
***Categorical variables***	**Individual training**		**Group training**		
	**N**	**%**	**N**	**%**	**Chi-square test**
Sex					
Male	99	36.4	65	28.8	*p =* 0.071
Female	173	63.6	161	71.2	
Education					
No formal education	26	9.6	17	7.5	*p =* 0.171
Incomplete primary	52	19.1	54	23.9	
Complete primary	122	44.9	104	46.0	
Secondary education	43	15.8	39	17.3	
University studies	29	10.7	12	5.3	
Civil status					
Single	17	6.3	11	4.9	*p =* 0.724
Married	177	65.1	141	62.4	
Widower	65	23.9	63	27.9	
Other type	13	4.8	11	4.9	
Frailty					
No	261	96.0	217	96.0	*p =* 0.972
Yes	11	4.0	9	4.0	
Polypharmacy					
No	117	43.0	100	44.2	*p =* 0.782
Yes	155	57.0	126	55.8	
Comorbidity					
No	68	25.0	50	22.1	*p =* 0.452
Yes	204	75.0	176	77.9	

*SD* Standard deviation

A total of 150 falls were recorded among 102 participants over the study period—59 in the first 6 months and 43 between months 6 and 12. Of the 102 participants, 29 had more than one fall.

### Primary outcome measure

Assessment of the equivalence between the interventions at 6 months showed that the confidence interval for the difference in people who reported at least one fall was within the equivalence limit of 10%, ∆ = -1.80 [-6.60 – 2.99]%. The people who reported at least one fall at 6 months was 11.03% for the individual training and 12.83% for the group training. During the evaluation at 12 months, we again found that the confidence interval for the difference in the people who reported at least one fall between treatments was within the equivalence limit of 10%, ∆ = -0.58 [-6.54 – 5.39]% (Table 2).

**Table 2:** Proportion of falls (number and percentage of participants who reported at least one fall) and number of falls (mean of falls)

	Individual training (*n =* 272)		Group training (*n =* 226)	
People who reported at least one fall	N	%	N	%
Baseline	78	28.7	70	31.0
12 months	55	20.2	47	20.8
**Number of falls**	**Mean**	**SD**	**Mean**	**SD**
Baseline	0.45	0.88	0.40	0.71
12 months	0.27	0.64	0.34	0.86

*SD* Standard deviation

We assessed the equivalence of the interventions separately for participants who had fallen or not in the previous year. In participants who had not fallen in the previous year the confidence interval for the difference in people who reported at least one fall was within the equivalence limit of 10% (falls at 6 months was 7.73% for individual training and 10.26% for group training; ∆ = -2.52 [-7.60 – 2.55]%; falls at 12 months was 15.46% for individual training and 17.95% for group training; ∆ = -2.48 [-9.08 – 4.11]%). However, in participants who had fallen in the previous year, the differences in falls exceeded the considered equivalence margin of 10% at 6 and 12 months. (falls at 6 months were 19.23% for individual training and 18.75% for group training; ∆ = 0.66 [-9.91 – 11.23]%; falls at 12 months were 32.05% for individual training and 27.14% for group training; ∆ = 4.91 [-7.38 – 17.20]%).

### Secondary outcome measures

#### People who reported at least one fall / Number of falls

Analysis of the people who reported at least one fall showed no differences between individual and group training (*p* = 0.571), although differences were observed between time points (OR= 0.596, 95% CI: 0.391-0.908), with a lower risk of suffering a fall after 12 months of intervention than in the previous year. No interaction was found between group and time (*p =* 0.786) (Table 2).

Analysis of the number of falls reported by study participants using the Poisson model revealed no differences between groups (*p =* 0.672), although differences were noted between time points (Incidence rate ratio (IRR) 0.713; 95% CI: 0.578 - 0.879), with a lower number of falls after 12 months of intervention than in the previous year. No interaction was found between group and time (*p =* 0.088) (Table 2).

#### Cause of falls

By analyzing the reasons for falls (bearing in mind that falls can occur for more than one reason), we found that the main cause of falls was the presence of an obstacle (32.6% of the reasons for falls at baseline and 34.8% during the intervention period) (Table 3).

**Table 3:** Cause of falls

		Baseline period		Follow up period	
	Group	N	%	N	%
Presence of an obstacle					
	IT	44	35.8	26	33.8
	GT	26	28.3	28	35.9
	Total	70	32.6	54	34.8
Get up/sit down/lying in bed or on the sofa					
	IT	4	3.3	7	9.1
	GT	4	4.3	1	1.3
	Total	8	3.7	8	5.2
Unsteady gait					
	IT	15	12.2	7	9.1
	GT	14	15.2	6	7.7
	Total	29	13.5	13	8.4
Poorly illuminated area					
	IT	7	5.7	3	3.9
	GT	6	6.5	4	5.1
	Total	13	6.0	7	4.5
Use of inappropriate footwear					
	IT	9	7.3	4	5.2
	GT	8	8.7	6	7.7
	Total	17	7.9	10	6.5
Slippery/wet/faulty floor					
	IT	33	26.8	25	32.5
	GT	29	31.5	23	29.5
	Total	62	28.8	48	31.0
Presence of clinical symptoms associated with falls					
	IT	11	8.9	5	6.5
	GT	5	5.4	9	11.5
	Total	16	7.4	14	9.0

*IT* Individual training, *GT* Group training

#### Consequences & assistance

Only one fall related to performing the Otago Exercise Programme exercises was identified—a minor transitory injury that did not require treatment—in group training. No differences were found during the intervention phase in terms of the reason for falls between the study groups (*p =* 0.378). There were no differences between groups in terms of the proportion of falls requiring treatment *(p =* 0.803) or in the proportion of falls with consequences (*p =* 0.179) (Table 4).

**Table 4:** Frequency and percentage of falls that received health care and falls with consequences

	Baseline period		Follow up period	
	N	%	N	%
Falls that required care				
IT	41	33.3	27	37.0
GT	27	30.0	30	39.0
Total	68	31.9	57	38.0
Falls that required care in primary healthcare centers				
IT	15	36.6	13	48.1
GT	12	44.4	14	46.7
Total	27	39.7	27	47.4
Falls requiring hospital care^a^				
IT	26	63.4	14	51.9
GT	15	55.6	16	53.3
Total	41	60.3	30	52.6
*Falls with consequences*				
IT	67	54.5	44	60.3
GT	48	53.3	38	49.4
Total	115	54.0	82	54.7

*IT* Individual training, *GT* Group training

^a^Emergency, emergency points or hospitalization

During the study, although there was a lower total number of falls, a greater proportion were treated (*p* < 0.001). We found no differences, however, in the number of falls with consequences, *p =* 0.264 (Table 4).

#### Adherence and satisfaction measures

Regarding the analysis of adherence, we found differences between the groups; the participants in the individual Otago Exercise Programme presented higher scores on the Exercise Adherence Rating Scale (EARS) test than did those in the group training (B=-1.384, *p =* 0.004). We also found poorer adherence at 12 months than at 6 months (B=-1.337, *p* < 0.001). We found no interaction between group and adherence, (*p =* 0.176). No differences were found in satisfaction between the groups at the end of the study (*p =* 0.585) (Table 5).

**Table 5:** Mean adherence and satisfaction

	IT (*n =* 272)		GT (*n =* 226)	
	Mean	SD	Mean	SD
Adherence				
Baseline	20.1	4.60	19.1	5.71
12 months	19.2	5.27	17.4	7.54
Satisfaction				
12 months	22.3	2.46	22.2	2.89

*IT* Individual training, *GT* Group training, *SD* Standard deviation

#### Dropouts

Of the total number of participants who completed the study (*n* = 498), study completion was greater among participants in individual training (*n =* 272, 64.9%) than among those in group training (*n =* 226, 55.4%), *p =* .005. The greatest loss of differential participants between groups occurred in participants who dropped out prior to the baseline assessment and before 6 months (for individual training, the losses occurred in 6.7% of participants prior to baseline, in 22.7% of participants before 6 months, in 3.8% of participants prior to 12 months and in 1.9% of participants due to other reasons; for group training, the losses occurred in 12% of participants prior to baseline, in 29.2% of participants before 6 months, in 2.9% of participants prior to 12 months and in 0.5% of participants due to other reasons). Despite the differential loss of participants, no differences were observed between the groups in demographic or clinical characteristics among those who did not complete the study (Table 6). Differences were observed only in the case of polypharmacy, where a greater number of people with polypharmacy in the individual training group dropped out.

**Table 6:** Differences between the groups among those who did not complete the study

	Individual training		Group training		Student’s t test
	Mean	SD	Mean	SD	
Age	72	4.5	72.3	4.5	*p =* 0.534
Weight	76.7	12.2	75.5	15	*p =* 0.498
Height	159.9	8.8	158.6	9.0	*p =* 0.233
BMI	29.9	4.1	30.0	5.6	*p =* 0.87
Cognitive	27.6	2.7	27.2	3.2	*p =* 0.32
Tinetti	31.9	3.1	31.0	3.8	*p =* 0.051
Fear of falling	21.9	6.1	22.5	7.1	*p =* 0.493
Barthel	98.6	3.7	98.4	3.4	*p =* 0.53
Lawton	7.7	0.7	7.5	1.1	*p =* 0.081
	**Individual training**		**Group training**		
	**N**	**%**	**N**	**%**	**Chi-square test**
Sex					
Male	47	32.0	57	31.3	*p =* 0.899
Female	100	68.0	125	68.7	
Education					
No formal education	17	11.6	20	11.0	*p =* 0.935
Incomplete primary	33	22.4	37	20.4	
Complete primary	70	47.6	86	47.5	
Secondary education	18	12.2	28	15.5	
University studies	9	6.1	10	5.5	
Civil status					
Single	4	3.0	13	8.4	*p =* 0.172
Married	76	57.1	92	59.7	
Widower	39	29.3	35	22.7	
Other type	14	10.5	14	9.1	
Frailty					
No	139	94.6	167	91.8	*p =* 0.322
Yes	8	5.4	15	8.2	
Polypharmacy					
No	62	42.2	102	56.0	*p =* 0.012
Yes	85	57.8	80	44.0	
Comorbidity					
No	47	32.0	70	38.5	*p =* 0.222
Yes	100	68.0	112	61.5	

*SD* Standard deviation

#### Intention-to-treat analysis

To assess the effect of participants who did not complete the study, differences in falls were analyzed for all those participants who had records of falls (805 at baseline and 531 at 12 months) independently of whether they had completed the study. We found no differences in the proportion of falls between groups (*p =* 0.765), although differences were found between time points (*p* < 0.001), with a lower proportion of falls after 12 months of intervention than in the previous year. We also did not find any interaction between group and time point (*p =* 0.971). In addition, analysis of the number of falls suffered by study participants showed no differences between the groups (*p =* 0.946), but differences were observed between time points (*p* < .001), with a lower number of falls after 12 months of intervention than in the previous year. We did not find any interaction between group or time point (*p =* 0.059).

## Discussion

This study has confirmed the hypothesis that group Otago Exercise Program is equivalent in effectiveness at reducing falls as individual format, at 6 and at 12 month follow up. Differences were not found between the proportion of falls in participants assigned to either individual or group Otago Exercise Programme or between group and time point. The same conclusions were reached with regard to the number of falls. The same conclusion was reached in the intention-to-treat analysis. However, when participant had had a previous fall in last year at baseline, group Otago Exercise Programme had potential to be more effective as equivalence was not within 10%. Evaluation of sample homogeneity revealed no statistically significant differences between group and individual Otago Exercise Program in terms of sociodemographic or clinical variables. In individual and group Otago Exercise Programme, 63.6% and 71.2% of participants were women and had a mean age of 71.6 and 72.1 years, respectively. The greater participation of women in clinical trials with exercises that assess reductions in falls is in line with the literature. According to the Cochrane Review [20], in terms of study characteristics in general, 77% of participants were women with a mean age of 76 years, which is greater than the mean age in our study. Although no studies with a similar design have assessed the efficacy of the Otago Exercise Programme in either format (group vs. individual) in the primary care setting were found, effectiveness data on the Otago Exercise Programme and other exercise programmes have been assessed in the literature. According to the Centers for Disease Control and Prevention compendium [10], fifteen exercise programs prevent falls. These programs involved, among other components, balance, strength endurance, flexibility and gait. Eleven of these studies were structured around groups, and four were individual interventions. In accordance with our study, the Cochrane Review [20] concluded that exercise interventions had demonstrated effectiveness whether they were carried out in a group or individual format. This review included four trials comparing group vs. individual exercise [12, 29–31]. The following conclusions were drawn from these studies: the rate of falls associated with injuries was 64% lower in the pilates group, although this difference was not statistically significant. In the large ProAct65+ study (three-arm, parallel-design cluster controlled trial), group exercise (FaME intervention) reduced falls after the intervention and at 12 month follow up after the intervention ceased, whereas individual exercise (Otago Exercise Programme) only showed a reduction in falls immediately post intervention [31]. This study, however, was comparing different interventions and the Otago Exercise Programme arm had poor adherence [31]. In a study that considered balance and functional outcomes (rather than falls), Otago Exercise Programme in a group format, or physical therapy delivered in groups, was more effective [30]. This study remains the first to consider the effect of Otago Exercise Programme in both formats on falls. Reductions in the proportion of fallers and rate of falls was similar to reviews of Otago Exercise Programme efficacy [32, 33].

When the proportions of falls in both groups were examined, differences were found between baseline and the 12-month follow-up, and these results were also obtained through analysis of the number of falls. This result is in line with findings in the literature. This program was initially tested in four randomized controlled trials and one controlled multicenter trial and showed its effectiveness in reducing falls. Overall, the fall rate was 35% lower among program participants than among those who did not take part [10]. According to the Cochrane systematic review on exercises for the prevention of falls [20], due to the abundance of solid evidence, exercise was found to reduce the rate of falls by 23%, and when balance and functional exercises were compared with controls, the rate was found to be reduced by 24%.

Regarding the characteristics of falls, the majority of falls resulted from the presence of an obstacle in both groups, followed by a slippery/wet/defective floor. This was no different to the reasons given at the baseline. One fall was recorded while the participants were exercising during group training. With respect to the treatment of falls, a significantly greater proportion of participants were treated but not in terms of consequences, with more falls treated in the healthcare center than in the hospital care setting from the basal phase to the study period, suggesting that the participants were encouraged to report all falls and attended the primary healthcare setting for follow-up. The Cochrane Review [20] revealed 27 RCTs of exercise; 14 reported no adverse events, and there were only two serious adverse events in one RCT (pelvic fracture & hernia surgery); the rest were nonserious adverse events, and there was a mean of 3 events in the group exercises.

The Otago Exercise Programme was delivered in a standardized manner with all deliverers trained and a progression protocol followed. The duration of the intervention was 12 months, following prior effective durations of Otago Exercise Programme [32, 33]. According to the Centers for Disease Control and Prevention compendium, in addition to the group vs. individual modalities, there is variability in the providers, number of sessions and duration [10]. The factor in common is that providers were certified or underwent standardized training, as in the present study. The duration ranged from 12 to 78 weeks. In a study that assessed adherence to the evidence-based recommendations of exercise programs to reduce falls in a community in Canada, variability between programs was found, indicating that the balance component was not well operationalized in practice with respect to challenge, documentation and progression [34]. This study did show a reduction in falls in both groups, suggesting fidelity to the protocols. According to a systematic review of long-term follow-up of exercise interventions aimed at preventing falls in older people living in the community, exercise interventions delivered over a longer duration, lasting from six months to a year, could reduce the rate and risk of falling by one-third [35]. The present study based its intervention on the original program and evidence following the implementation manual [19]. The programmed face-to-face training sessions were in weeks 1, 2, 4 and 8, with reinforcement at 6 months in the primary healthcare center.

Each participant received audiovisual and written support material with instructions for all the prescribed exercises along with the necessary equipment to perform the muscle strengthening exercises (weights). To encourage adherence to the supervised sessions and the home exercise in between, monthly reminders were issued when there was no face-to-face follow-up with regard to the intervention, and adherence and satisfaction were assessed in each of the groups. According to the World Health Organization, adherence is the measure in which the behavior of a person – taking medication, following a diet and/or carrying out lifestyle changes – corresponds to the recommendations agreed upon by a healthcare provider [36]. However, there are differences in the studies on the definition of adherence and inclusion of adherence with acceptance and a gap in the literature on well-established measures [37]. Many of these refer to attendance at sessions, while others refer to acceptance of the program. This adherence is measured as a percentage of participation, without a clear consensus on what percentage represents “good adherence”, and very few studies have examined exercise intensity [38]. An appropriate, consistent record could facilitate the implementation of preventive activities [39]. A systematic review of long-term follow-up of exercise interventions aimed at preventing falls in older people living in the community concluded that it was not possible to evaluate finer-level details, such as the extent of participant attendance, adherence to or quality of prescribed exercise individually or in group-delivered sessions [35]. In our study, we did not consider attendance at sessions, either individually or in groups, as a measure of adherence but rather of exercise at home at least twice per week. For this purpose, we used the EARS scale, which has been validated to measure adherence to the prescription of exercise at home [24, 40, 41]. Although not explicitly analyzed in our study, we know that peer support, socialization, social networks, perceived health benefits and professional guidance, among other factors, have been identified as key motivators of program adherence [42, 43]. Nonetheless, our study revealed that individuals in the training group presented higher scores on the EARS test than did those in the training group, which indicated greater adherence, despite no different in outcomes. Additional objective measures are required to determine to what extent this occurs. Linking exercise to activities of daily living can improve adherence by including the movements learned in training sessions at home and increasing awareness of the benefits to one’s autonomy. We also found lower adherence at 12 months than at 6 months, and this result may be related to the face-to-face reinforcement provided up to that month. The reduction in adherence over time is in agreement with data reported in other studies [44]. In the systematic review by Ashworth et al., brief programs showed greater effectiveness in terms of physical variables when delivered in healthcare centers than when carried out at home. However, in long-term programs, home training programs have shown better adherence [45]. There was no difference in satisfaction between the groups. Considering the equivalence seen in this study in terms of outcomes of falls, and the dropouts at baseline suggesting preferences for format delivery, health professionals should offer a choice of either group or individual formats for delivery of Otago Exercise Programme to increase uptake of this evidence based exercise program.

### Limitations

The project protocol identified the need to perform analyses by both protocol and intention to treat. The number of losses was greater than estimated given that any variation in the experimental conditions established meant the loss of a study participant even though the intervention was maintained in clinical practice. Due to the study design, the intention-to-treat analysis yielded the same results as the per-protocol analysis. There were no statistically significant differences between the clinical and sociodemographic variables of the groups that dropped out of the study, with the exception of polypharmacy, which was greater in the individual training group.

## Conclusion

The group Otago Exercise Programme is equivalent to individually delivered Otago Exercise Programme in terms of prevention of falls over a 12-month follow-up. The Otago Exercise Programme is globally effective at reducing falls, irrespective of format. Self-reported adherence was greater in the individual training group, while there were no statistically significant differences in self-reported satisfaction in either group, suggesting either format could be offered depending on patient preference or service abilities.

## Supplementary Information

Supplementary Material 1.

## Data Availability

Patient’s booklets produced during the research project will be available in the following repository: https://repisalud.isciii.es/. Availability of data is not applicable because request and permission of third parties are required.
